# *Cutibacterium acnes* lysate improves cellular response against *Candida albicans*, *Escherichia coli* and *Gardnerella vaginalis* in an *in vitro* model of vaginal infection

**DOI:** 10.3389/fcimb.2025.1578831

**Published:** 2025-05-02

**Authors:** Francesco Ricchi, Samyr Kenno, Natalia Pedretti, Giulia Brenna, Francesco De Seta, Andrea Ardizzoni, Eva Pericolini

**Affiliations:** ^1^ Clinical and Experimental Medicine PhD Program, University of Modena and Reggio Emilia, Modena, Italy; ^2^ Department of Surgical, Medical, Dental and Morphological Sciences with Interest in Transplant, Oncological and Regenerative Medicine, University of Modena and Reggio Emilia, Modena, Italy; ^3^ Department of Medical Sciences, University of Trieste, Trieste, Italy; ^4^ Department of Obstetrics and Gynecology, Istituto di Ricovero e Cura a Carattere Scientifico (IRCCS) San Raffaele Scientific Institute, University Vita and Salute, Milano, Italy

**Keywords:** vulvovaginal candidiasis (VVC), bacterial vaginosis (BV), aerobic vaginitis (AV), bacterial lysates, cutibacterium acnes, Candida albicans, Gardnerella vaginalis, Escherichia coli

## Abstract

**Introduction:**

The vagina is a finely balanced microecological environment. The rupture of this balance leads to dysbiosis, which causes the resident microbiota to be overcome by pathogens. This event triggers the onset of gynecological infectious diseases, normally treated with antimicrobial drugs, considered to date as the gold standard; yet the increasing rate of drug resistance requires novel approaches and alternative therapeutic strategies. Bacterial lysates, obtained by mechanical or chemical crushing of the bacterial cell walls, contain several antigens and Pathogen-Associated-Molecular-Patterns (PAMP) molecules that through the priming of epithelial and innate immune cells could improve the responses to the pathogens.

**Materials and methods:**

We evaluated the effect of a bacterial lysate (BL) obtained by *Cutibacterium acnes* on the response of a human vaginal epithelial cell line and a murine macrophage cell line to the infection by *C. albicans*, *E. coli* and *G. vaginalis*. By priming the cells with BL, the mitochondrial Reactive Oxygen Species (mtROS) production, the cellular damage, the impairment of microbial growth, the phagocytic and killing capacity and the secretion of cytokines and chemokines were assessed.

**Results:**

BL did not show any direct antimicrobial effect nor any toxicity for the cell lines employed. Upon infection with *C. albicans*, the BL-primed cells were shown to increase the production of mtROS, to be more resistant to pathogen-mediated cell damage, and to reduce the microbial growth. BL-primed macrophages displayed also an increased phagocytic and killing activity against *C. albicans, E. coli* and *G. vaginalis.* Cytokines and chemokines secretion by BL-primed vaginal epithelial cells was also modulated upon infection with *C. albicans*, *E. coli* and *G. vaginalis*.

**Discussion:**

Overall, the results shown here point to the possible role of BL in priming epithelial and phagocytic cells and to improve their response against bacterial and fungal pathogens. These data indicate that the use of this (and, in future, other bacterial lysates) may provide a promising novel approach to handle lower genital tract infections through the reinforcement of local immunity.

## 1 Introduction

The vagina of healthy women during the reproductive age is colonized by many microbial species, mainly *Lactobacillus* spp. (up to 95%) (Ravel et al., 2011). The healthy vaginal microbiota helps the host to keep pathogens at bay through mechanisms of competitive inhibition, production of bacteriocins and lactic acid and by stimulating the host cells to release antimicrobial peptides and anti-inflammatory cytokines (Niu et al., 2017; Ilhan et al., 2019). The microecological balance of the vagina is a finely tuned dynamic process, capable of self-regulation. The breaking up of such balance leads to dysbiosis, with the resident microbiota overcome by pathogens, triggering the onset of gynecological infectious diseases (Chao et al., 2019; Zhang et al., 2021). Vulvovaginal candidiasis (VVC), bacterial vaginosis (BV) (and to a lesser extent aerobic vaginitis, AV) are the most common infections of the lower genital tract.

VVC is a symptomatic inflammation of the vagina, that affects 70-75% of healthy women at least once during their reproductive age (Blostein et al., 2017; Denning et al., 2018). It is caused by several species of the genus *Candida*, mainly by *C. albicans*. VVC is mainly endogenous because *C. albicans* is a member of the human microbiota and it dwells in the mucosae of the oropharynx, genital and gastrointestinal tracts (Odds, 1987; Kauffman, 2006; Pappas, 2006; Pfaller and Diekema, 2007). In the context of VVC the infection and disease frequently occur in healthy women and to date a higher incidence of the disease has not been described under conditions of immunodepression. However, some studies indicated increased susceptibility in specific categories (i.e., diabetics and immunocompromised women) (Talaei et al., 2017; O’Laughlin and McCoy, 2023). Even though *Candida* causes damage *per se*, the host response plays an important role in VVC onset, by exacerbating the fungal-mediated damage and causing the symptoms, which include itching, burning, pain, redness of the vulva and vaginal mucosa and a typically thick and white (cottage-cheese-like) vaginal discharge (Ardizzoni et al., 2021).

Bacterial vaginosis (BV) is a condition characterized by a modification of the bacterial milieu, where beneficial healthy vaginal microbiota is dramatically reduced and overwhelmed by facultative and strictly anaerobic pathogens, such as *Gardnerella* spp., *Prevotella* spp., *Peptostreptococcus* spp., *Mobiluncus* spp., *Atopobium vaginae* and *Mycoplasma hominis* (Srinivasan and Fredricks, 2008; Turovskiy et al., 2011; Chen et al., 2021). It affects women during their fertile age, it is characterized by specific clinical symptoms (thin, homogeneous and grayish-white vaginal discharge, rotten fish vaginal odor, and less commonly itching and burning sensation) and it is accompanied by serious obstetrics and gynecologic complications: spontaneous abortion, preterm birth, endometritis, pelvic inflammatory disease, postoperative infections (Jacobsson et al., 2002; Leitich et al., 2003; Rothman et al., 2003; Guerra et al., 2006; Kavoussi et al., 2006) and increased susceptibility to sexually transmitted diseases (Brotman, 2011).

Aerobic vaginitis (AV) is a less common vaginal infection, where the normal vaginal microbiota is overcome by aerobic bacteria often deriving from the Gastro-Intestinal (GI) tract, such as *Escherichia coli*, *Streptococcus agalactiae*, *Staphylococcus aureus* and *Enterococcus faecalis* (Donders et al., 2002, 2011, 2017; Kaambo et al., 2018). These bacteria can become dangerous, especially during pregnancy, because not only do they affect fetal health, but the pregnancy status aggravates the symptoms and the consequences of the infection (Donders et al., 2017; Fan et al., 2021; Plisko et al., 2021). The etiology and pathogenesis of AV are not completely clear (Fan et al., 2021; Mohankumar et al., 2022). The pathogenic bacteria are retained to produce different toxins or, similarly to VVC, to affect the local immunity of patients, thus leading to the disease onset (Ma et al., 2022). AV symptoms are viscous and yellow vaginal discharge, sticky fishy odor, stinging and burning sensations and dyspareunia (Donders et al., 2017).

To date, antibiotics (metronidazole, clindamycin and tinidazole) and antifungal drugs (fluconazole, amphotericin B, nystatine, flucytosine) are the gold standard treatments for BV/AV and VVC, respectively (Gaziano et al., 2020). However, given the ever-growing problem of drug resistance, it is necessary to develop novel therapeutic strategies as an aid or an alternative to the current pharmacological approach.

The alteration of vaginal mucosa integrity is a key step in the pathological process of lower genital tract infections. For example, *G. vaginalis* can disrupt the epithelial barrier integrity and alter the immune microenvironment in the vaginal tract (Rahman et al., 2024). Preservation of the integrity and functionality of the epithelial barrier is crucial to counteract infections, hence the potentiation of epithelial cells play a pivotal role against pathogens.

In recent years, the emerging concept of “trained immunity” modified the idea of memory responses that traditionally were a prerogative only of the adaptive branch of the immune system (Netea et al., 2016). Indeed, increasing data show that also the cells of the innate immunity, as well as the epithelial cells, are able to acquire a memory phenotype through the modulation of epigenetic, metabolic and functional changes in response to several stimuli of infectious (Liu et al., 2016; Kaufmann et al., 2018; Bigot et al., 2019; Covián et al., 2019, 2021), and non-infectious nature (Bekkering et al., 2014). It has been demonstrated that following these changes, such cells can increase cytokines production and antigen presentation, ultimately improving the antimicrobial responses. The basis of this phenomenon has been reconducted to the presence of several Pathogen Associated Molecular Pattern (PAMP) molecules that trigger the trimethylation of the lysine residue in position 4 on the histone H3 (H3K4me3). This chemical modification facilitates the transcription of several genes, especially those coding for proinflammatory cytokines (Acevedo et al., 2021). Epigenetic and metabolic reprogramming act on epithelial cells by improving their mechanical barrier, enabling them to modulate cytokines production and stimulating them to produce alarmins and other antimicrobial peptides. They act also on innate immune cells, such as macrophages and NK cells, by increasing their mitochondrial activity, triggering the inflammasome, and modulating cytokines production and their phagocytic activity (Chalifour et al., 2004; Zhang and Mosser, 2008). By taking advantage of the new knowledge of the mechanisms of trained immunity, new approaches have been introduced to potentiate the response of the host, such as the use of bacterial lysates. Bacterial lysates are obtained by mechanical or chemical crushing of the bacterial cell walls (Bizzini et al., 1984; Pfefferle et al., 2013), and the fragments obtained contain several antigens and PAMP molecules. Literature reports the successful employment of bacterial lysates to prevent recurrent respiratory diseases, to avoid the flare-up of respiratory chronic infections and as a defense against urinary infections (Braido et al., 2007; Ahumada-Cota et al., 2020).

*Cutibacterium acnes* (*C. acnes*) (formerly known as *Propionibacterium acnes*) lysate proved effective for the treatment of type 1 hypersensitivity caused by soaps, solvents, chemicals and cosmetics (Mangano et al., 2017). Because this species normally dwells on the skin, it is prone to several kinds of stressful stimuli and therefore it is endowed with as many defense systems. In particular, *C. acnes* is equipped with the enzyme radical oxygenase that allows the bacteria to reduce the reactive oxygen species (ROS), thus counteracting the oxidative stress (Allhorn et al., 2016). One particular fraction of the *C. acnes* lysate, the P40, has been previously demonstrated to be effective for the treatment of chronic obstructive bronchitis as well as recurrent infections of the genitourinary tract from *C. albicans*, *E. coli* and Herpes Simplex Virus (Jurkiewicz and Zielnik-Jurkiewicz, 2018; Triantafillou et al., 2019; Yang et al., 2019). The present study aims at evaluating the activity of the *C. acnes* bacterial lysate (BL) in *in vitro* models of microbial infections from the fungus *C. albicans*, the Gram- variable bacterium *G. vaginalis* and the Gram-negative bacterium *E. coli*.

## 2 Materials and methods

### 2.1 Cells, microorganisms and bacterial lysate

#### 2.1.1 Cells

The human vaginal epithelial cell line A-431(ATCC-CRL-1555) and the murine macrophages J774A.1 cell line (ATCC-TIB-67) employed in this study were purchased from American Type Culture Collection. Cells were cultured, as previously described, with slight modifications (Spaggiari et al., 2023). Briefly, both cell lines were cultured in Dulbecco’s Minimum Essential Medium (DMEM) (Sial S.r.l., Rome, Italy), supplemented with heat-inactivated fetal bovine serum (Hi FBS Sial) at 10% (vol/vol) in the growth medium and 5% (vol/vol) in the maintenance medium, 100 U/ml Penicillin-Streptomycin (Lonza Walkersville Inc., Walkersville, MD, U.S.A.), 2.5 mg/ml Ciprofloxacin (Gibco, Thermo Fisher Scientific Italia) and 2 mM L-glutamine (Gibco).

#### 2.1.2 Microbial strains and growth conditions

All the microorganisms employed in the present study had been stocked frozen at -80°C in Microbank^®^ cryovials (ProLab Diagnostics, Richmond Hill, ON, Canada). After thawing, the reference strains *C. albicans* SC5314 (ATCC MYA-2876) and *C. parapsilosis* CLIB214 (ATCC 22019) were grown in Yeast Extract Peptone Dextrose (YEPD) broth (Condalab, Madrid, Spain) and subcultured in Sabouroud Dextrose Agar (SDA – Oxoid, Thermo Scientific Italia) through weekly passages. *E. coli* (ATCC 13762) was grown in Tryptic Soy Broth (TSB – Biolife S.r.l., Milano, Italy) and subcultured in MacConkey agar (BioChemika, Sigma-Aldrich, Germany) through weekly passages. *G. vaginalis* (ATCC 14018) was grown in New York City III (NYC III) broth (ATCC Medium 1685) and *L. crispatus* (ATCC 33280) was grown in De Man, Rogosa Sharpe (MRS) broth (Oxoid). All the microorganisms were used in their exponential growth phase for the infection assays (see below). *C. albicans* and *C. parapsilosis* were subcultured in SDA and *E. coli* was subcultured in TSB; the incubations were carried out for 24 h at 37°C under agitation (120 rpm) and in aerobic conditions. For both *L. crispatus* and *G. vaginalis*, an aliquot from the frozen stock was placed in broth and cultured at 37°C under agitation (120 rpm) and in anaerobic conditions for 24 h and 48 h respectively.

#### 2.1.3 Bacterial lysate

Bacterial lysate (BL) was prepared from *C. acnes* (ATCC 6919) as described elsewhere (Ohashi et al., 1983; Basal et al., 2004). Whole cells, suspended in an appropriate volume of distilled water, were disrupted through ultrasonication. The mixture was then centrifuged at 2,200 × g for 20 min to remove any intact cells. The resulting supernatant underwent further centrifugation at 20,000 × g for 30 min. The obtained pellet was treated with pronase at 80°C for 2 h. Following another centrifugation at 20,000 × g for 30 min, the final pellet was lyophilized and designated as the cell wall fraction. After the preparation of the lyophilized cell wall fraction, a mother solution at 10 mg/ml in dimethyl-sulfoxide (DMSO) was prepared. Then, BL was used at 100, 10 or 1 μg/ml, diluted in the respective media. In all the experiments, the respective dilution of DMSO was included, as internal control, and it did not show any effect.

### 2.2 Effect of BL on fungi and bacteria by MIC determination

The broth microdilution method was employed to evaluate the possible direct effect of BL on pathogens viability. Specifically, BL starting from the concentration of 0.160 mg/ml was serially diluted 1:2 in 96-well, flat-bottomed plates in Roswell Park Memorial Institute (RPMI) 1640 (Sigma Aldrich) added with glucose 18 g/l (Carlo Erba, Milano, Italy) and 4-Morpholinapro-pansulphonic acid (MOPS, 35 g/l) (Sigma Aldrich) for *Candida* and *E. coli*. The broth microdilution method was carried out for *G. vaginalis* by using NYC III broth and for *L. crispatus* by using MRS broth. Subsequently, 100 μl of the microorganism (5x10^5^ CFU/ml for all microorganisms except for *G. vaginalis* and *L. crispatus*, 5x10^6^ CFU/ml) were added to each well. The growth of the microorganisms was assessed by measuring the optical density (OD) with a spectrophotometric plate reader (Sunrise, Tecan, Männedorf, Switzerland), 24 h and 48 h after inoculation. Readings were performed at 540 nm (for fungi) and 595 nm (for bacteria) wavelengths. Each species, seeded in its specific medium without bacterial lysate and diluent, served as a positive control.

### 2.3 Effect of BL on A-431 and J774A.1 cells by flow cytometry analysis

A-431 and J774A.1 cell cultures (1x10^5^ cells/well) were grown for 24 h or 2 h, respectively, in 96-well plates. Then, cells were washed with 200 µl of warm PBS to remove dead cells and antibiotic residues. Next, 100 μl of 10-fold serially diluted BL in maintenance medium (with or without antibiotics) were added in triplicate to the cell cultures and incubated for a further 24 h. A-431 cells were then detached by Trypsin-EDTA solution (0,25%) in HBSS (1×) (GIBCO) whereas J774A.1 cells were then detached with a cell-scraper. Cells were then centrifuged for 10 min at 4°C at 350 × g and the supernatants were harvested. Next, the cells were stained with eBioscience™ Fixable Viability Dye eFluor™ 780 (Thermo Fisher Scientific, U.S.A.), diluted in BSA/PBS 0.5% w/vol, to discriminate live cells from dead cells. Each sample was incubated for 15 min in ice, in the dark. Next, the cells were centrifuged for 10 min at 4°C at 350 × g, the supernatant was discarded, and the pellet was resuspended in Fixation Buffer (BioLegend, U.S.A.) and incubated for 20 min in the dark at room temperature. Each sample was centrifuged one last time for 10 min at room temperature at 350 × g, the supernatant was discared, and the pellet was resuspended in 350 µl of PBS for analysis. The FACSymphony™ (Becton Dickinson, U.S.A.) was employed and the software FlowJo™ was used for the data analysis. Heat-killed (HK) A-431 and J774A.1 cells were included as internal controls.

### 2.4 A-431 and J774A.1 cell assays

**Figure 1:**
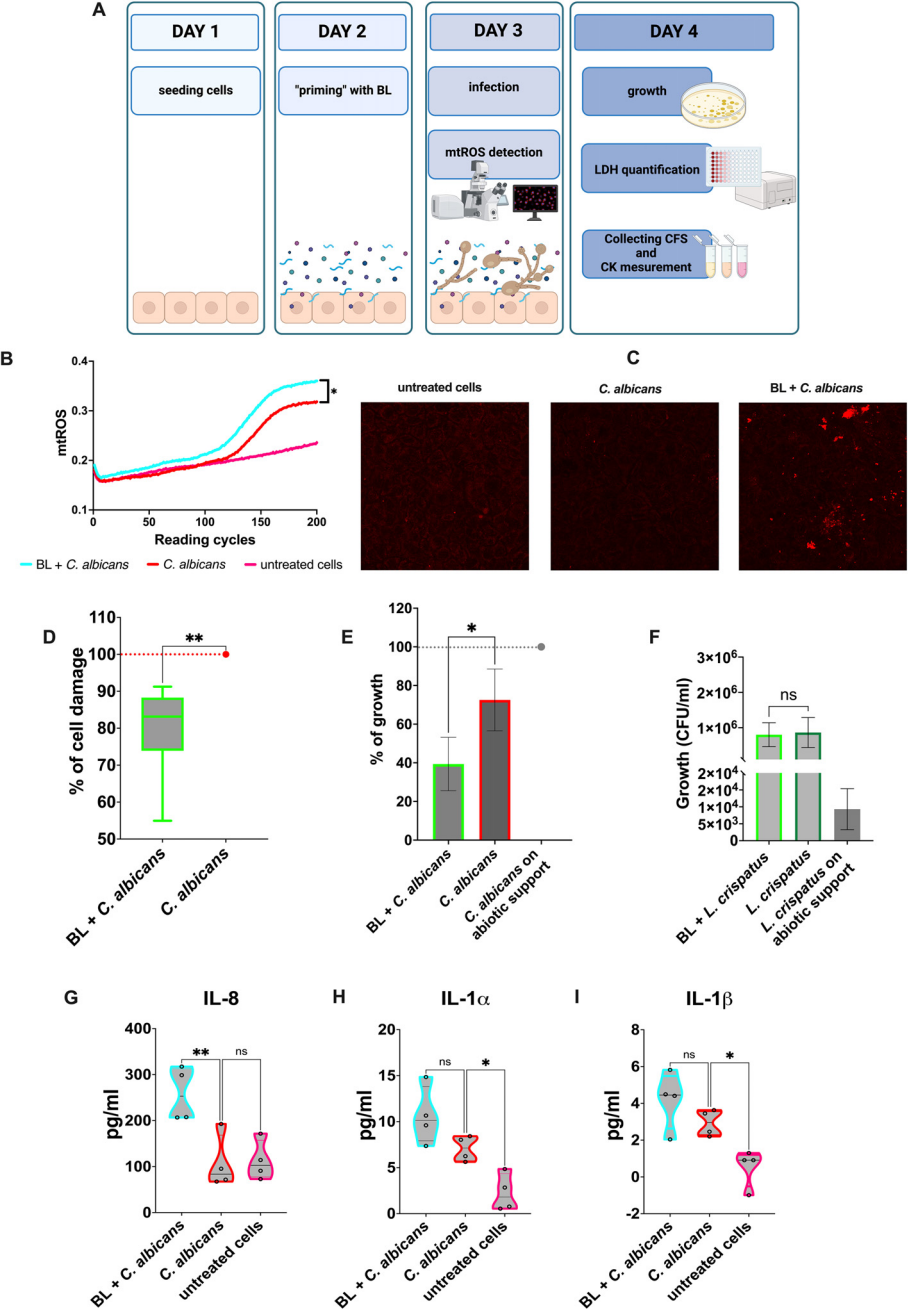
Effects of BL priming on A-431 cells infected with *C albicans*. **(A)** Schematics representing the treatment and the experiments carried out (created with BioRender.com). **(B)** Kinetic quantification of mtROS production by infected A-431 cells primed or not with BL (10 μg/ml). Data are from 3 experiments performed in triplicate **(C)** Visualization of mtROS production in A-431 cells primed or not with BL (10 μg/ml) by confocal microscopy, 30 min after infection with *C albicans*. **(D)** Percentage of damage in BL-primed (1 μg/ml) and infected A-431 cells. The boxplot results from six experiments conducted in triplicate. **(E, F)** Effect of BL-primed (1 μg/ml) A-431 cells on the growth of *C albicans* **(E)** and *L. crispatus* **(F)** determined through Colony Forming Units (CFU) counts; the data are expressed as the mean ± SD of at least 3 experiments. **(G–I)** IL-8 **(G)**, IL-1β **(H)** and IL-1α **(I)** production by BL-primed (10 μg/ml) infected A-431 cells. Each truncated violin results from 4 experiments. The values of *p < 0.05 and **p < 0.01 were considered statistically significant. ns, not significant.

**Figure 2:**
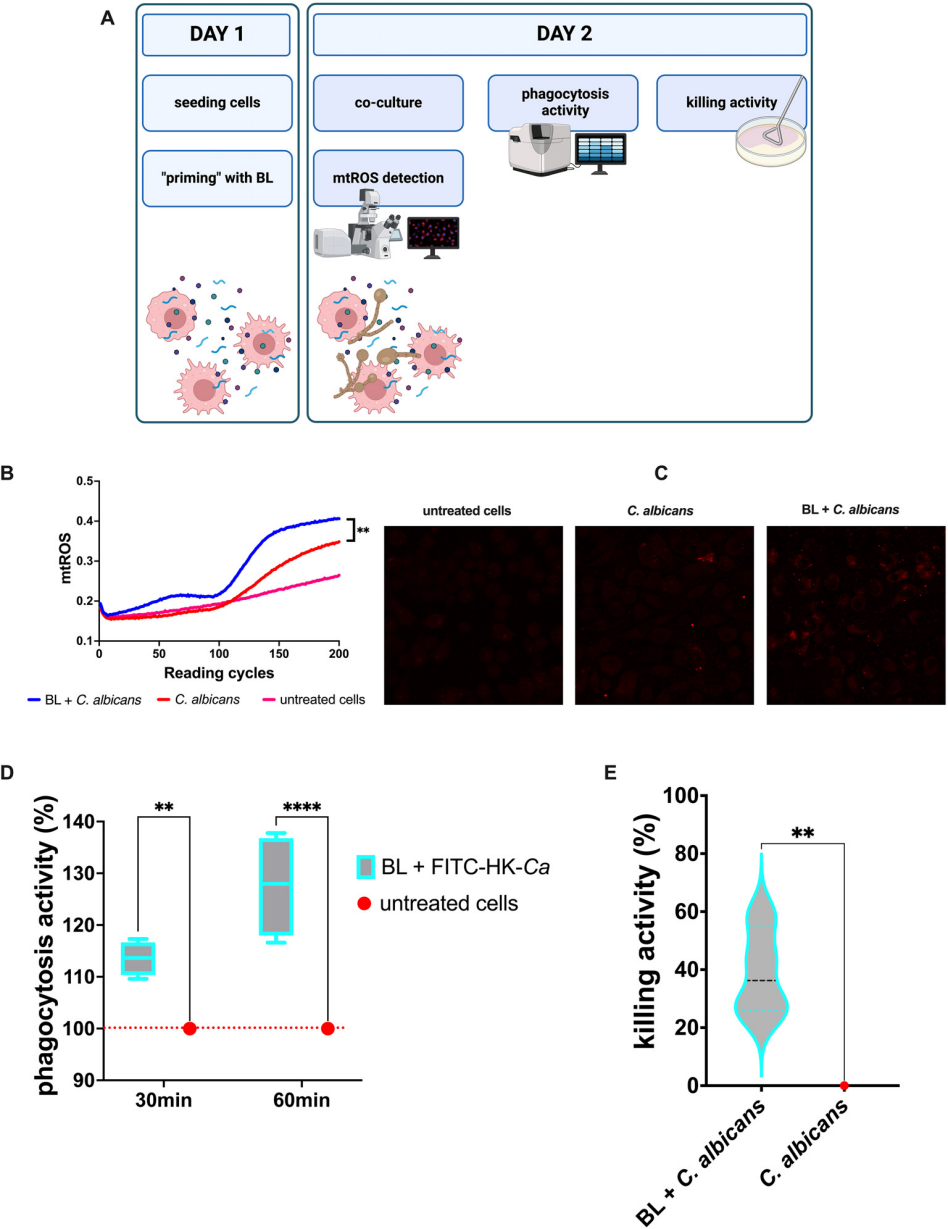
Effects of LB priming on J774A.1 cells infected with *C albicans*. **(A)** Schematics representing the treatment and the experiments carried out (created with BioRender.com). **(B)** Kinetic quantification of mtROS production by BL-primed (100 μg/ml) infected J774A.1 cells. Data are from 3 experiments performed at least in duplicate. **(C)** Visualization of mtROS production in J774A.1 cells primed or not with BL (100 μg/ml) by confocal microscopy, 30 min after infection with *C albicans*. **(D)** Percentage of phagocytic activity of BL-primed (10 μg/ml) J774A.1 cells after 30 and 60 min of coculture with FITC-labelled heat-killed *C albicans* (FITC-HK-Ca). Each boxplot results from 4 experiments conducted in triplicate. **(E)** Percentage of killing activity by BL-primed (10 μg/ml) J774A.1 cells against *C albicans*. Data results from four experiments conducted in duplicate. The values of **p < 0.01 and ****p < 0.0001 were considered statistically significant.

The assays performed on the A-431 cells are depicted in **Figure 1A**. Briefly, 24 h after seeding (at day 1), the A-431 cells were primed by removing the growth medium and adding BL into the fresh medium (day 2). At day 3 the BL-primed A-431 cells were infected with *C. albicans* or the bacteria and the production of mtROS in response to the infection was kinetically evaluated. Finally, at day 4 after seeding, the growth of microorganisms, the A-431 cell-damage and cytokines and chemokines production in response to the infection were assessed. All the assays performed on the J774A.1 cells are depicted in **Figure 2A**. Briefly, the J774A.1 cells were seeded and primed with BL (day 1). At day 2 the BL-primed J774A.1 cells were infected with *C. albicans* or the bacteria and the production of mtROS in response to the infection was kinetically evaluated. Finally, at day 2 after seeding, the phagocytosis and killing activity of the BL-primed J774A.1 were assessed.

#### 2.4.1 Evaluation of microorganisms-induced cell damage by lactate-dehydrogenase quantification

Serially diluted BL was added to cell cultures grown for 24 h, as described in paragraph 2.3. Finally, the infection was performed at different MOI (Multiplicity of infection, defined as the ratio between the number of cells and the number of microorganisms). One hundred µl of each microorganism suspension, in maintenance medium, was added. Specifically, MOI 1:5 was used for *C. albicans*, MOI 1:1000 was employed for *E. coli*, whereas for *G. vaginalis* a MOI 1:1 was applied. The plate was then placed again for 24 h at 37°C with 5% CO_2_. Cytotoxicity was quantified by analyzing the LDH release in the culture medium, by employing a commercially available kit (Roche, via Sigma-Aldrich) following the Manufacturer’s instructions. The percentage of damage was calculated as follows:


$$\%\;of\;cell\;damage=\frac{sample-\;low\;control}{\;high\;control-\;low\;control}\;\times 100$$

where: low control is the average value of uninfected cells and high control is the average value of uninfected cells lysed with 1% (vol/vol) Triton-X-100 (Fluka). Then, the data were expressed as percentages, assuming as 100% the damage of infected cells not primed with BL.

#### 2.4.2 Evaluation of microorganisms’ growth after coculture with BL-primed epithelial cells

BL was added to cell cultures grown for 24 h, as described in the previous paragraph. *C. albicans* (MOI 1:5) or *L. crispatus* (MOI 1:50) were then added to the cells. After 24 h of culture, the cells were detached with 0.2% (vol/vol) Tryton-X-100. The samples were collected in 1.5 ml tubes. All the wells were then washed with 100 μl of Soybean–Casein Digest Broth prepared with Lecithin and Polysorbate 80 (SCLDP 80) medium (Biotec) to completely detach the fungi and the bacteria, and the detached microorganisms were added to the respective tubes. Next, the tubes were centrifuged at 3,500 rpm for 5 min, the supernatants were discarded, and the pellets were resuspended with 1 ml of PBS. Finally, serial dilutions were performed and seeded onto SDA plates (*C. albicans*) or MRS plates (*L. crispatus*). The data were expressed as percentages, assuming as 100% the maximum growth of *C. albicans* in wells not containing cells. Normalization was not performed on *L. crispatus*, and its growth was expressed as CFU/ml.

#### 2.4.3 Kinetic quantification of mtROS in BL-primed and *C. albicans* infected cells

For the determination of mtROS production, A-431 cells were seeded on black-transparent 96 well plates, infected by *C. albicans* at MOI 1:5 and then 2.5 μM/well MitoSOX™ Red (Invitrogen™, Thermo-Fisher Scientific) were added immediately to each well. Then, the fluorescence intensity was measured in kinetics (1 reading cycle every 5 min) by means of a Fluoroskan FL microplate fluorometer (Thermo Scientific, Waltham, MA, U.S.A.) under stable temperature of 37°C. The fluorescence emission was analyzed at excitation/emission wavelengths of 544 nm/590 nm, according to an established protocol (Spaggiari et al., 2024). The assay was carried out under the same experimental conditions also for J774A.1 cells but using a different MOI (1:10). Confocal microscopy was employed for the visualization of mtROS in A-431 and J774A.1 cells. The cells were seeded at a density of 5 x 10^5^ cells per compartment in a 4-compartments cell culture dish (Greiner Bio-One, Italy). Imaging was then performed using a confocal microscope (Leica SP8 confocal microscope equipped with 405-nmand white light lasers) and the resulting images were analyzed using Fijii software (ImageJ).

#### 2.4.4 Quantification of cytokines production in BL-primed infected cells

The secretion of IL-1α, IL-1β, TNF-α and IL-8 by BL-primed A-431 cells 24 h after infection with *C. albicans*, *E. coli* and *G. vaginalis* was assessed, using the same MOI described above (see paragraph 2.4.1). The detection of cytokines and chemokines was achieved by using commercial ELISA kits (PeproTech™, ThermoFischer Scientific, Cranbury, NJ, USA for IL-1α and TNF-α; Invitrogen™, Thermo-Fisher Scientific for IL-1β and IL-8), which were used according to the Manufacturers’ instructions. The analyses were carried out on the supernatants that had been collected after LDH quantification or from *ad-hoc* experiments carried out with the same protocol used for LDH detection and stored at -20°C.

#### 2.4.5 Phagocytosis and killing activity of BL-primed J774A.1 cells

*C. albicans*, subcultured for 24 h on SDA, was resuspended in PBS at the working concentration of 1x10^9^ CFU/ml. The fungal cells were then inactivated by heating them at 90°C for 30 min. Next, the killed yeasts were labelled with 0.1 mg/ml fluorescein 5(6)-isothiocyanate (FITC) (Sigma Aldrich) for 15 min at room temperature, in the dark and occasionally flipping. The labelled heat-killed (HK) *Candida* cells were then aliquoted and kept as frozen stocks at -20°C. The FITC stock solution was prepared at 1 mg/ml in 0.05 M carbonate-bicarbonate buffer. Cell cultures (1x10^5^ cells/well) were grown for 2 h in a black-transparent 96-well plates. Then, the growth medium was removed and 100 μl of 10-fold serially diluted BL in maintenance medium were added to the cell cultures and incubated for a further 24 h. Next, 100 µl of 1x10^6^ FITC-labeled HK *Candida* suspension in maintenance medium were added to each well, and the multiwell plate was incubated for further 30 min and 60 min. After these incubation times, the medium was removed and 100 μl of 0.4% (w/vol) Trypan Blue solution (Corning, U.S.A.) were added; then, the plate was further incubated for 1 min. Finally, the Trypan Blue was removed, and the fluorescence emission was analyzed by Fluoroskan FL microplate fluorometer at excitation/emission wavelengths of 490 nm/521 nm. The percentage of phagocytosis was calculated as follows:


$$\%\;of\;phagocytosis=\frac{sample-\;blank\;control}{\;untreated\;cells-\;blank\;control}\;\times 100$$

where blank control is represented by average fluorescence value of medium with FITC labelled HK *Candida* and the untreated cells represents the average level of cell phagocytosis. To evaluate the killing activity of BL-primed J774A.1 cells, the growth medium was removed, and serially diluted BL was added to cell cultures that had been grown for 2 h in growth medium with or without antibiotics, according to the microorganism assessed. Then, the cells were infected with *C. albicans*, *E. coli* and *G. vaginalis*, all employed with MOI 1:1. After 4 h of infection with *C. albicans* or 2 h of infection with the bacteria, the cells were detached with 0.2% (vol/vol) Tryton-X-100 and the samples were collected in 1.5 ml tubes. Control samples consisting of microorganisms growth in the wells without cells were detached with SCDLP80. Next, serial dilutions were performed and seeded onto SDA plates for *C. albicans*, Tryptic Soy Agar (TSA, Condalab, Spain) for *E. coli*, and *Gardnerella vaginalis* agar (Microbiol, Uta, CA, Italy) for *G. vaginalis.* All the plates were incubated at 37°C with 5% CO_2_. The percentage of killing activity was calculated as follows:


$$\%\;of\;killing\;activity=100-\frac{\;CFU\;sample\;\times \;100}{\;CFU\;m.o.\;on\;abiotic\;support}$$

where “CFU m.o. on abiotic support” is represented by the average number of CFU of the microorganism grown under the same conditions, without cells. The percentage of killing increase was then calculated as follows:


$$\%\;of\;killing\;increase=\frac{\%\;of\;killing\;sample\;\times \;100\;}{\%\;of\;killing\;of\;untreated\;cells}-100$$

where “% of killing of untreated cells” represents the basal killing activity of the untreated cells, infected with the pathogen.

### 2.5 Statistical analysis

The Shapiro-Wilk test was used to analyze data distribution within each experimental group. Subsequently, statistical analysis was performed by one-way ANOVA or the Kruskal Wallis test, depending on the distribution of data; Dunnett’s multiple comparisons or Dunn’s test respectively were chosen as *post-hoc* tests. For the kinetic curve obtained in the mtROS assessment procedures, the Area Under the Curve (AUC) was calculated to summarize the curve into a single value. Subsequently, statistical analysis was performed on the AUC values of each experimental group using one-way ANOVA. Don’t corrected test for multiple comparison was chosen as a *post-hoc* test. To analyze the data of the phagocytosis activity in terms of dose and infection time, a two-way ANOVA test, followed by Dunnett’s multiple comparisons, were used. All statistical analyses were carried out using GraphPad Prism 10 software. Values of * p < 0.05, ** p < 0.01 and **** p < 0.0001 were considered statistically significant.

## 3 Results

### 3.1 Effect of BL on microorganisms

By employing the broth microdilution method, the direct effect of BL on fungi (*C. albicans* and *C. parapsilosis*) as well as on bacteria (*E. coli*, *L. crispatus* and *G. vaginalis*) was evaluated after 24 h and 48 h of incubation. The results show that BL did not impair the growth of any of the microorganisms, regardless of BL concentrations (0.16 mg/ml to 0.63 µg/ml), as shown in the charts of **Supplementary Figure S1**.

### 3.2 Effect of BL on A-431 and J774A.1 cells viability

In order to establish the lack of toxic effects of BL on the vaginal epithelial cell line A-431 and on the murine phagocytic cell line J774A.1, a cytofluorimetric analysis was carried out to quantify the percentage of live cells after the treatment with BL. As shown in **Supplementary Figure S2**, BL did not affect the viability of epithelial (**Supplementary Figure S2A**) and phagocytic (**Supplementary Figure S2B**) cell lines, regardless of its concentrations.

### 3.3 A-431 vaginal epithelial cells primed with BL increased mtROS production, reduced cell damage, impaired microbial growth and modulated cytokines and chemokines secretion in response to *C. albicans* infection

A-431 vaginal epithelial cells responded to *C. albicans* infection by increasing the mitochondrial activity as a defense mechanism against the fungus. Such an increase was achieved through the production of mitochondrial ROS (mtROS), whose level was much higher than that of the untreated cells. Interestingly, when the vaginal cells had been primed with BL prior to being infected by *C. albicans*, the mtROS production was significantly higher compared to vaginal cells that were not subjected to such pretreatment (**Figure 1B**). Representative microscopy images showed an increased production of mtROS in BL-primed cells after 30 min of *C. albicans* infection, as compared to *C. albicans* infected cells (**Figure 1C**). The production of higher levels of mtROS suggests that epithelial cells primed with BL could respond more efficiently to the fungal pathogen. Not only did BL treatment make A-431 cells more responsive against the pathogen, but it caused them to become also more resistant to the fungal-induced cell damage. Indeed, A-431 cells primed with BL prior to fungal infection, significantly reduced the levels of LDH release, a marker of cell damage, with respect to the control cells (**Figure 1D**). In addition, the effect of BL on A-431 cells caused indirect damage to the fungus. Indeed, *C. albicans* recovered after the infection of BL-primed A-431 cells grew significantly less than *C. albicans* recovered from A-431 un-primed cells (**Figure 1E**). It is worth noting that such an effect could not be observed when A-431 cells were colonized with *L. crispatus*, a species considered beneficial for the vaginal tract. This *Lactobacillus* could grow efficiently after recovering from both BL-primed and un-primed A-431 cells (**Figure 1F**). Moreover, when primed with BL, infected A-431 cells significantly increased IL-8 secretion with respect to un-primed infected cells (**Figure 1G**). The secretion of IL-1α (**Figure 1H**) and IL-1β (**Figure 1I**) in primed infected cells resulted slightly increased, but the levels of both these cytokines remained comparable to those of *C. albicans* un-primed infected cells. Finally, the assessment of TNF-α levels returns cytokine levels below the detection limit in response to *C. albicans* infection, irrespective of the pretreatment with BL (data not shown).

### 3.4 J774A.1 murine macrophages primed with BL increased mtROS production, phagocytosis activity and killing capacity in response to *C. albicans* infection

As for the vaginal epithelial cell line, the J774A.1 murine macrophages responded to *C. albicans* infection by increasing the oxidative burst, which for this kind of cell is one of the main defense mechanisms against microbial pathogens. Similarly to the results observed for the vaginal epithelial cells, in the BL-primed and infected macrophages the levels of mtROS were significantly higher than in the un-primed *C. albicans* infected cells (**Figures 2B**). Representative microscopy images showed an increased production of mtROS in BL-primed cells after 30 min of *C. albicans* infection, as compared to *C. albicans* infected cells (**Figure 2C**). This result strongly suggests that BL has all the potential to improve macrophages’ antimicrobial activity. Indeed, the priming with BL significantly increased the phagocytosis activity of J774A.1 of FITC labelled heat-inactivated (HK) *C. albicans* after 30 min (and even more after 60 min) of coculture (**Figure 2D**). In addition, the percentage of killing activity of J774A.1 cells against *C. albicans* was significantly higher when the macrophages were primed with BL, with respect to the un-primed cells (**Figure 2E**).

### 3.5 A-431 vaginal epithelial cells primed with BL reduced cell damage and modulated cytokines and chemokines secretion in response to bacterial infections

**Figure 3:**
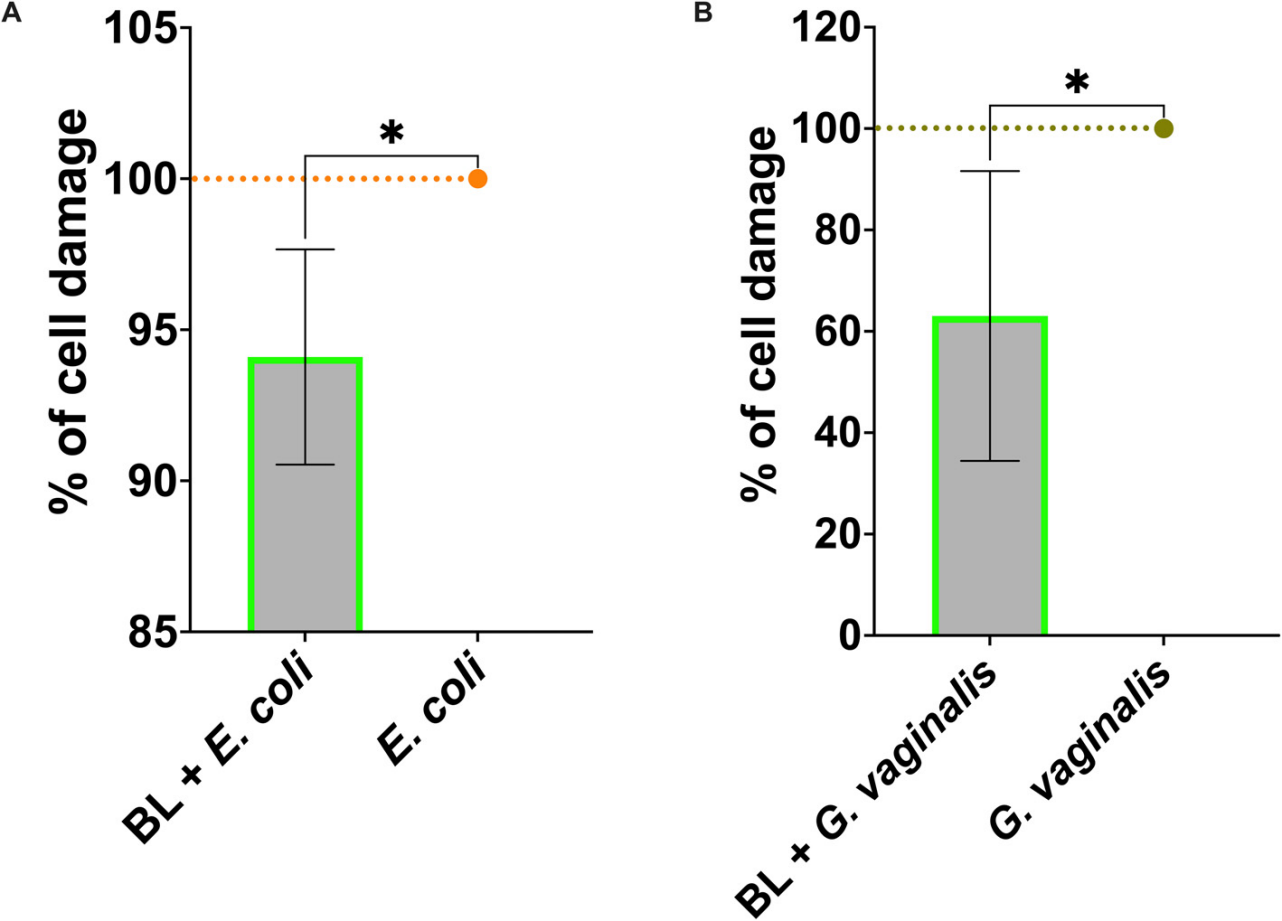
Effect of BL priming on A-431 cell damage induced by *E coli* **(A)** and *G vaginalis* **(B)**. Percentage of cell damage of BL-primed (1 μg/ml) and infected A-431 cells. The data are expressed as the mean ± SD of at least 3 experiments. The values of *p < 0.05 were considered statistically significant.

**Figure 4:**
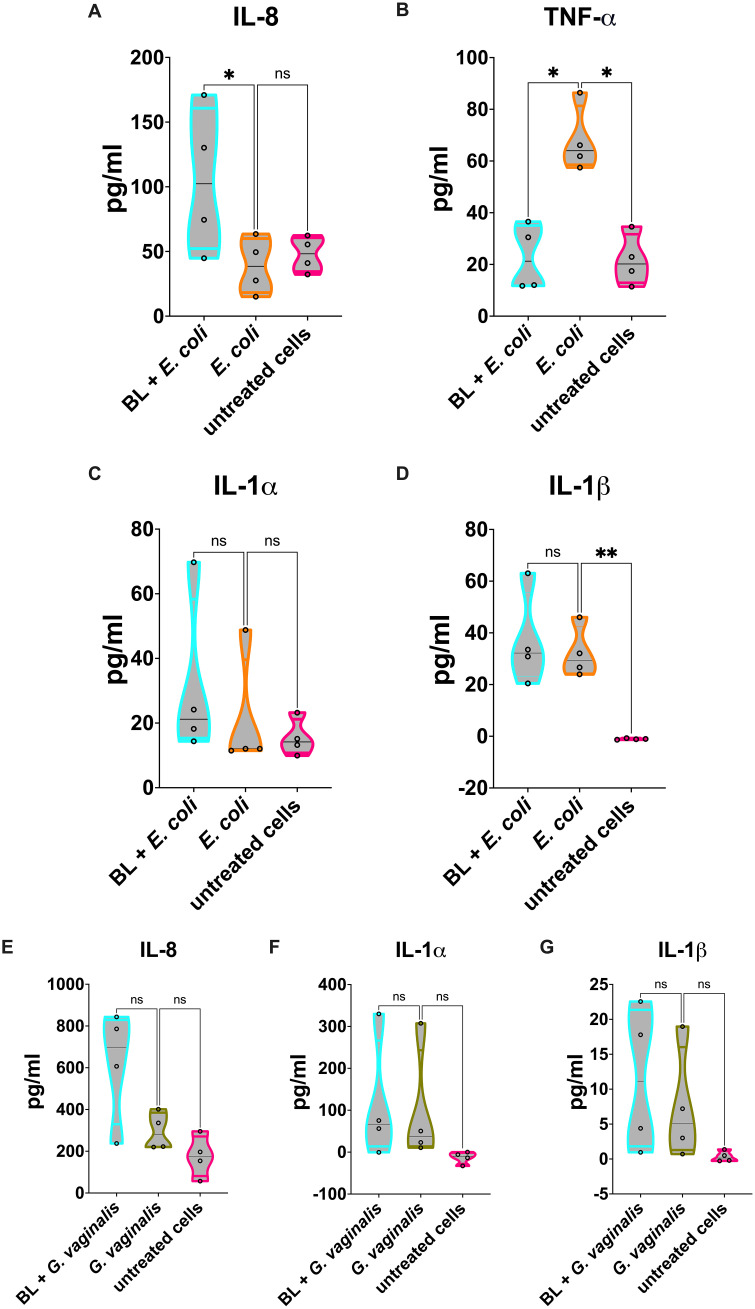
Cytokines and chemokines released by BL-primed A-431 cells infected with *E coli* or *G vaginalis*. **(A-D)** IL-8 **(A)**, TNF-α **(B)**, IL-1α **(C)** and IL-1β **(D)** production by *E coli* infected A-431 cells primed or not with BL (10 μg/ml). **(E-G)** IL-8 **(E)**, IL-1α **(F)** and IL-1β **(G)** production by *G vaginalis* infected A-431 cells primed or not with BL (10 μg/ml). Each truncated violin results from 4 experiments. The values of *p < 0.05 were considered statistically significant, ** p < 0.01. ns, not significant.

Similarly to what was observed in *C. albicans* infection, our results show a significant reduction of cell damage in the vaginal epithelial cells primed with BL and infected with *E. coli* (**Figure 3A**) or *G. vaginalis* (**Figure 3B**). Following *E. coli* infection, a significant increase of IL-8 (**Figure 4A**) was observed in BL-primed cells. Differently, the levels of the inflammatory cytokine TNF-α were significantly reduced in BL-primed cells in response to *E. coli* infection, reaching levels similar to those secreted by the untreated cells (**Figure 4B**). Finally, the levels of IL-1α (**Figure 4C**) and IL-1β (**Figure 4D**) did not increase when the infected cells had been primed with BL, and the levels of both these cytokines remained comparable to those of *E. coli* infected un-primed cells. Upon A-431 cells infection with *G. vaginalis*, the levels of IL-8 (**Figure 4E**), IL-1α (**Figure 4F**), and IL-1β (**Figure 4G**) did not change, irrespective of the pretreatment or not with BL, even though a trend of increased IL-8 could be observed in BL-primed as compared to un-primed infected cells (**Figure 4E**). Differently from *E. coli*, TNF-α levels returns cytokine levels below the detection limit in response to *G. vaginalis* infection, irrespective of the pretreatment with BL (data not shown).

### 3.6 J774A.1 cells primed with BL increased killing activity against *E. coli* and *G. vaginalis*

**Figure 5:**
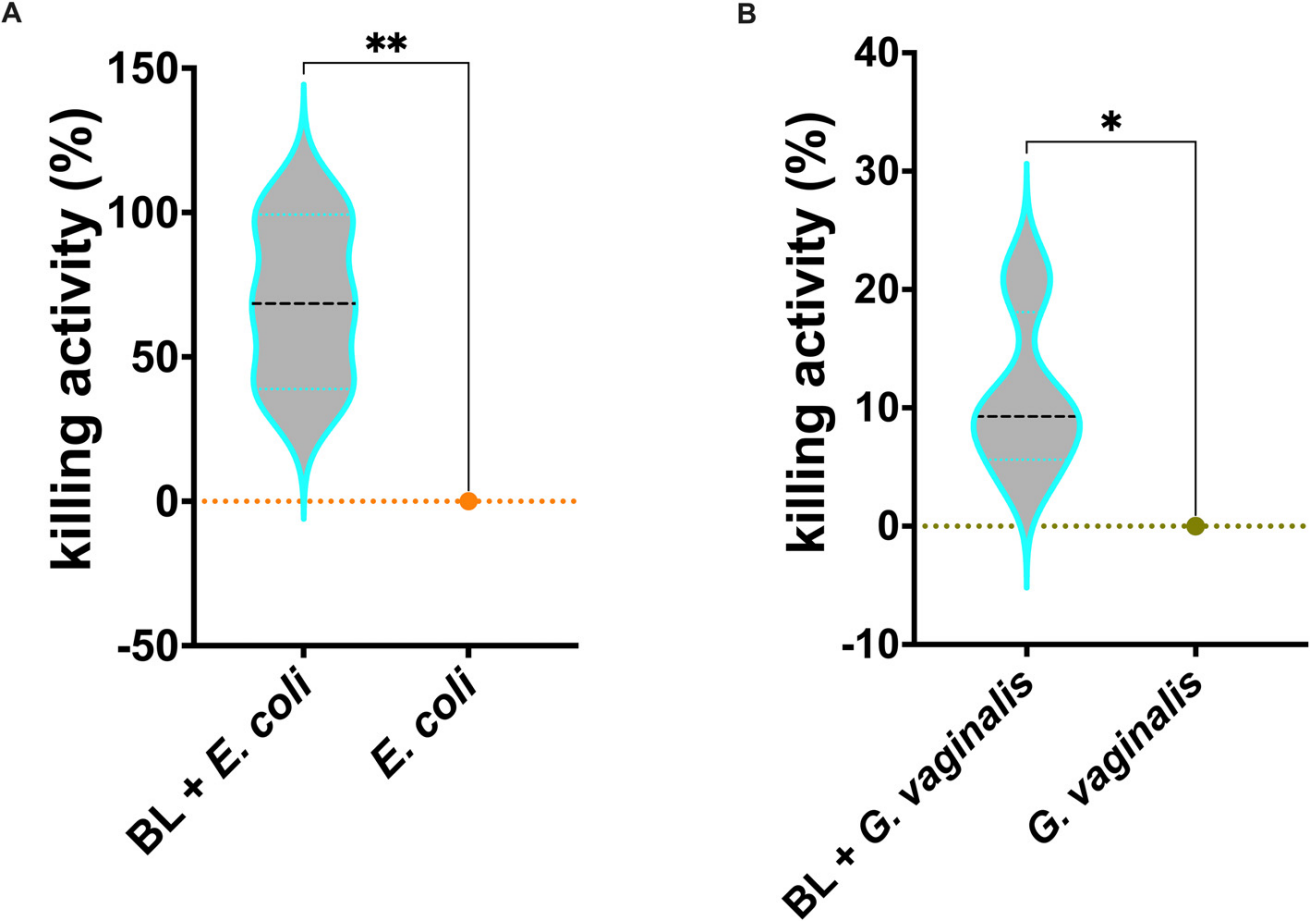
Effects of BL priming on J774A.1 cells infected with *E coli* **(A)** or *G vaginalis* **(B)**. Percentage of BL-primed (10 μg/ml) J774A.1 killing activity against *E coli* and *G vaginalis*. Data result from at least 3 experiments conducted in duplicate. The values of *p < 0.05 and **p < 0.01 were considered statistically significant.

Similarly to what was observed for *C. albicans* infection, the killing activity of J774A.1 macrophages primed with BL was significantly increased against both *E. coli* (**Figure 5A**) and *G. vaginalis* (**Figure 5B**) as compared to un-primed infected cells.

## 3 Discussion

*Candida albicans* is a normal commensal of the human body. It dwells on the skin and on the mucosal surfaces of oro-pharynx, gastro-intestinal and female genital tract. When the immune system is compromised, *Candida* shifts from commensalism to opportunism, becoming a pathogen and causing disease. Interestingly, in the context of VVC the infection and disease are not necessarily associated with the immune system impairment; indeed, VVC frequently affects healthy women. However, some studies indicated increased susceptibility in specific categories, such as diabetics and immunocompromised women (Talaei et al., 2017; O’Laughlin and McCoy, 2023). Furthermore, it has been demonstrated that even though *Candida* causes damage *per se*, the host response plays an important role in VVC onset, by exacerbating the fungal-mediated damage and causing the symptoms, which include itching, burning, pain, redness of the vulva and vaginal mucosa and vaginal discharge (Ardizzoni et al., 2020, 2021).

Bacterial vaginosis (BV) is characterized by alterations in the vaginal environment and a shift in the vaginal microbiota from *Lactobacillus* species to a high bacterial diversity, including facultative anaerobes. *Gardnerella* spp. dwells in the vagina of patients with BV and it and it represents the key bacteria in the pathogenesis of BV (Salinas et al., 2020). Aerobic vaginitis (AV), has an incidence ranging from 2.0 to 25.8% (Donders et al., 2009), and the associated pathogenic microorganisms are mainly *Escherichia coli*, *Enterococcus* spp., *Streptococcus angina*, and *Streptococcus agalactiae*.

The treatment with antifungal drugs (the gold standard, to date) is accompanied by the risk of developing drug resistance. Similarly, in both BV and AV, the antimicrobial agents are widely used. However, resistance to these agents is the major cause of recurrent vaginitis. Therefore, the definition of novel therapeutic strategies is warranted as an alternative to the current pharmacological approach.

By using *in vitro* models of microbial infection, here we have evaluated the protective activity of the *C. acnes* bacterial lysate (BL) against the most common lower genital tract pathogens.

In order to establish whether BL acts by improving epithelial and innate immune cells response to the infections, without exerting a direct effect on vaginal microorganisms, we have performed experiments where bacteria and fungi have been incubated with a range of BL dilutions for 24 or 48 h. The lack of any effect demonstrates that BL at least in our *in vitro* system, does not exert any direct antimicrobial activity, thus suggesting that in an eventual therapeutic treatment it should not affect the resident microbiota. However, we assessed the effect of BL only on 5 microorganisms. A broader analysis covering more strains and species is warranted to support our results. Then, to exclude a possible toxic effect of BL, epithelial and phagocytic cell lines have been treated or not with BL at different concentrations, and the percentage of alive cells has been assessed. The results demonstrate the absence of any toxicity of BL at least under our experimental conditions, thus suggesting that it might be well tolerated by human cells. Therefore, the response to the infections by BL-primed cells is very likely to occur mainly through mechanisms involving the improved reactivity of vaginal epithelial cells and macrophages against microbial pathogens. Among such responses, the increase of mtROS plays a crucial role in innate immunity (West et al., 2011). In line with this phenomenon, both our *in vitro* models (A-431 vaginal epithelial cells and J774A.1 macrophages) show an increase in mtROS production upon infection with *C. albicans* in BL-primed cells. In line with our recent results, demonstrating that mtROS is a key element of vaginal epithelial cells response to *C. albicans* (Spaggiari et al., 2024), the data reported here show that not only does the BL make the cells capable of responding better to an infectious insult, but it makes them also less prone to being damaged by fungal and bacterial pathogens. This is evident by the significantly lower levels of cell damage in BL-primed vaginal epithelial cells infected with *C. albicans*, *E. coli* and *G. vaginalis*. By strengthening the vaginal epithelial cells, BL also counteracts microbial growth, albeit indirectly. Indeed, *C. albicans* retrieved from BL-primed vaginal epithelial cells have grown significantly less than *C. albicans* retrieved from the un-primed cells. Interestingly, such effect has not been observed upon colonization with the beneficial microbe *L. crispatus*, thus strengthening the idea that BL “instructs” the vaginal epithelial cells to respond specifically only to harmful microorganisms.

The improvement of vaginal epithelial cells performance passes also through the modulation of cytokines and chemokines release in response to the infection. In particular, the BL-primed A-431 cells produce significantly higher levels of IL-8, upon infection with *C. albicans* and *E. coli* and increased (albeit not significant) levels of IL-8 upon infection with *G. vaginalis*. In addition, significantly lower levels of TNF-α following *E. coli* infection have been observed. IL-8 is a chemokine also known as “neutrophils chemotactic factor” because it induces chemotaxis in neutrophils and in other granulocytes, causing them to migrate to the site of infection. Notably, previous clinal studies demonstrate that the absence of leukocytes in most women with BV is likely due to the lack of IL-8 induction (Cauci et al., 2002, 2003; Cauci, 2004). Should this BL-induced enhancement of IL-8 secretion be demonstrated also *in vivo*, it would imply that this lysate could play an active role in the establishment of the innate immune response. TNF-α is a cytokine that plays a central role in the inflammatory responses, by inducing either survival or death in target cells. The levels of this cytokine are reported to increase in AV, but not in VVC (Hedges et al., 2006; Kalia et al., 2019). Consequently, upon *E. coli* infection of BL-primed A-431 vaginal epithelial cells, the reduction of TNF-α suggests BL may have an immunomodulatory role, but more evidence is needed to directly link this to AV treatment.

The effect of BL cell priming has been assessed also on macrophages, which are typical cells involved in innate immune responses. As shown by the results presented here, the effects of BL on the phagocytic cells are not limited to the increase of mtROS. Indeed, BL-primed J774A.1 cells significantly improve their phagocytic activity against HK *C. albicans*. In addition, BL priming makes the macrophages more effective in their killing activity with respect to un-primed J774A.1, and such killing capacity has been shown to increase significantly against *C. albicans*, *E. coli* and *G. vaginalis*. These results strengthen our idea that BL-primed immune cells are more responsive to microbial pathogens. Overall, the results shown here point to the possible role of BL in priming epithelial and phagocytic cells to improve their response against bacterial and fungal pathogens. Such effects should be assessed also on other microorganisms or even viruses relevant for lower genital tract infections. These data indicate that the use of this (and, in future, other bacterial lysates) may provide a promising novel approach to handle lower genital tract infections through the reinforcement of local immunity. It should be pointed out that while *C. acnes* lysate (and possibly other microbial lysates) may be a promising novel approach for the management of lower genital tract infections, further *in vivo* and clinical studies are warranted to confirm its efficacy.

## Data Availability

The original contributions presented in the study are included in the article/**Supplementary Material**. Further inquiries can be directed to the corresponding author.
