# Supplementary material for: Cutibacterium acnes lysate improves cellular response against Candida albicans, Escherichia coli and Gardnerella vaginalis in an in vitro model of vaginal infection
Source: Front Cell Infect Microbiol. 2025 May 2;15:1578831. doi: 10.3389/fcimb.2025.1578831 (PMC12081444; doi:10.3389/fcimb.2025.1578831)
Supplement: Supplementary file 1 [file DataSheet1.docx]

**Figure S1. Effects of scalar doses of BL on microorganisms’ viability.** The effect of serially diluted BL (0.16 mg/ml to 0.63 µg/ml) on microorganisms’ viability was assessed after 24 h (left panels) and 48 h (right panels) contact with *C. albicans* (**A**), *C. parapsilosis* (**B**), *E. coli* (**C**), *L. crispatus* (**D**) and *G. vaginalis* (**E**). Data are expressed as mean ± SD of 3 different experiments.
