# Supplementary material for: Cutibacterium acnes lysate improves cellular response against Candida albicans, Escherichia coli and Gardnerella vaginalis in an in vitro model of vaginal infection
Source: Front Cell Infect Microbiol. 2025 May 2;15:1578831. doi: 10.3389/fcimb.2025.1578831 (PMC12081444; doi:10.3389/fcimb.2025.1578831)
Supplement: Supplementary file 2 [file DataSheet2.docx]

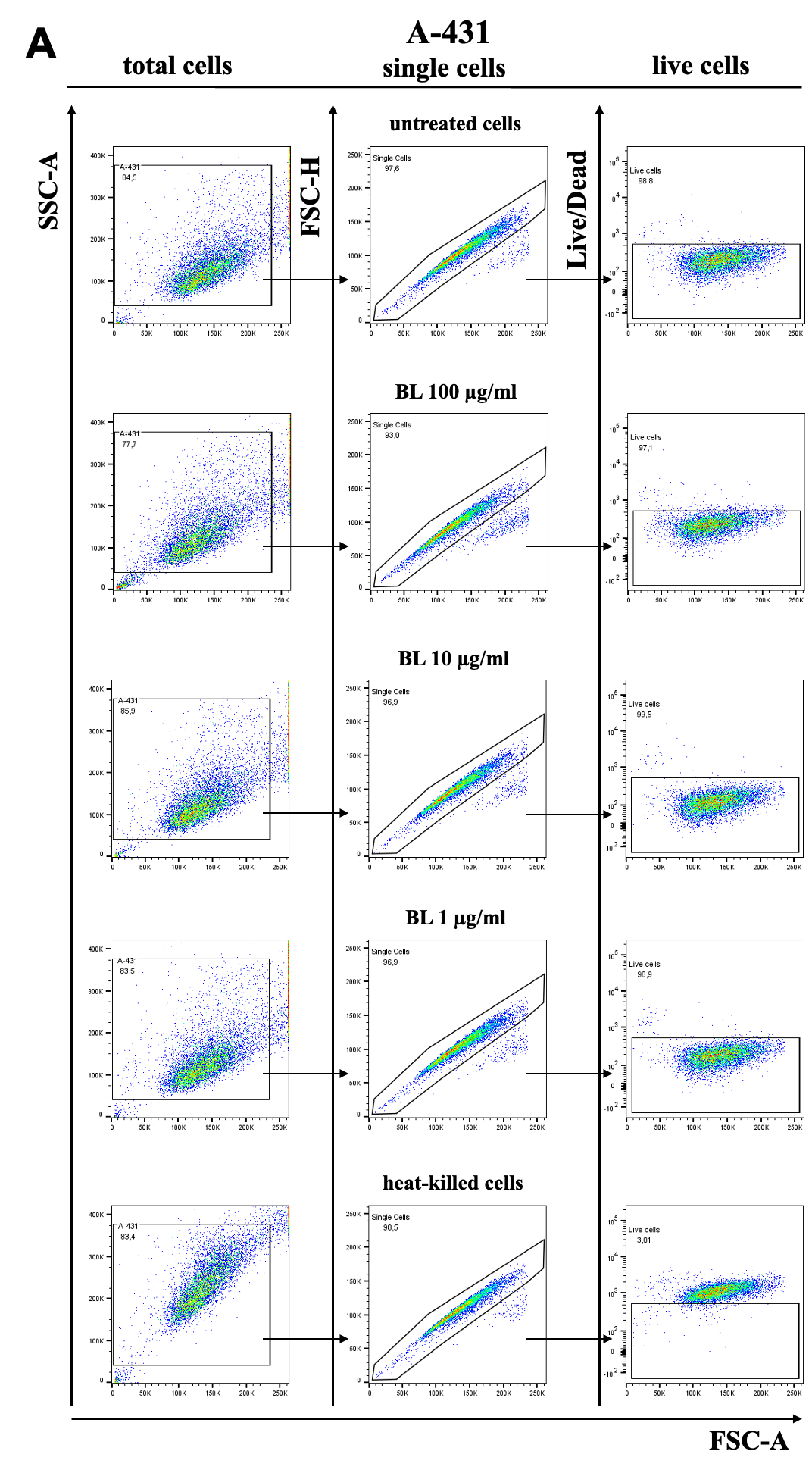


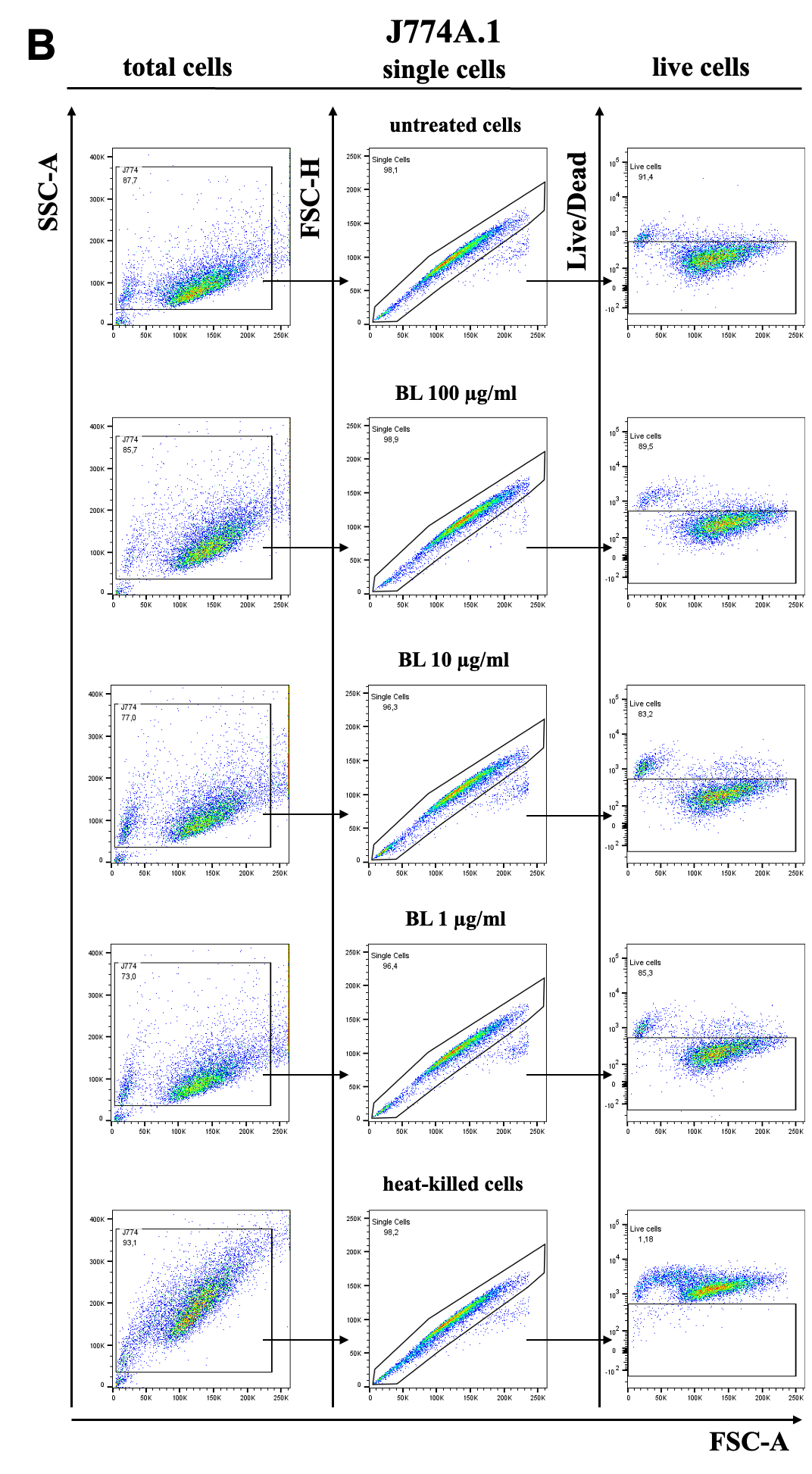


**Figure S2. Effect of BL on cells viability.** Effect of serially diluted BL (100, 10 and 1 µg/ml) on the viability of A-431 cells (A) and J774A.1 macrophages (B) was assessed by cytofluorimetric analysis. Heat-killed cells were used as positive controls, whereas untreated cells were included as negative controls. Cytofluorimetric plots with the gating strategy are shown.
